# From Exposure to Effect: Genetic and Epigenetic Biomarker-Guided Risk Assessment in Cardiac Imaging

**DOI:** 10.3390/ijms27073041

**Published:** 2026-03-27

**Authors:** Andrea Borghini, Francesca Gorini, Mariangela Palazzo, Jalil Daher

**Affiliations:** 1 National Research Council, Institute of Clinical Physiology, 56124 Pisa, Italy; francesca-gorini@cnr.it (F.G.); mariangelapalazzo@cnr.it (M.P.); 2 Department of Biology, Faculty of Arts and Sciences, University of Balamand, Balamand 100, Lebanon; jalil.daher@balamand.edu.lb

**Keywords:** ionizing radiation, cardiac imaging, interventional cardiology, γ-H2AX, micronucleus, telomeres, circulating-cell free DNA, mitochondrial dysfunction, DNA methylation, microRNA

## Abstract

The rapid expansion of cardiac imaging has substantially increased patient and occupational exposure to low-dose ionizing radiation. Evidence suggests that cumulative exposures below 100 mSv may contribute to long-term risks of cancer and non-cancer diseases, including cardiovascular disease. However, establishing causality at these dose levels is challenging, as epidemiological studies are limited by heterogeneous endpoints, uncertainties in dose reconstruction, and incomplete control of confounding factors. Molecular biomarkers offer a promising strategy to bridge the gap between radiation exposure and clinically manifest disease, enabling more precise individualized risk assessment and targeted preventive strategies. This review summarizes current evidence on genetic and epigenetic biomarkers for evaluating the biological effects of radiation in cardiac imaging and interventional cardiology and examines their potential role in risk stratification and occupational surveillance. Genetic markers—including γ-H2AX foci, micronucleus assays, and telomere length alterations—alongside epigenetic modifications such as DNA methylation changes and microRNA expression profiles provide sensitive indicators of radiation-induced cellular damage. Integrating biomarker profiling with individualized dosimetry and longitudinal follow-up may improve risk prediction, enhance occupational protection, and support safer, more sustainable imaging practices in contemporary cardiovascular care.

## 1. Introduction

Exposure to ionizing radiation (IR) from environmental, occupational, and medical sources is associated with potential adverse effects on human health. Increasing evidence indicates that low-dose IR—typically defined as less than 100 mSv—can contribute to the long-term risk of cancer and cardiovascular disease [1,2]. Low-dose ionizing radiation from cardiac imaging is associated with a small but significant increased risk of cancer and cardiovascular disease. While individual test risk is low, cumulative exposure, especially in sensitive populations, increases the long-term risk of malignancies and vascular issues [3].

In cardiovascular medicine, these concerns have prompted major professional societies to emphasize the appropriate and justified use of medical radiation, balancing diagnostic and therapeutic benefits against potential long-term risks [4]. Medical imaging procedures represent the largest source of man-made radiation exposure in developed countries, with cardiac imaging contributing substantially due to high procedural volumes and relatively high dose per examination [5]. Beyond radiological protection considerations, the expanding use of cardiac imaging has also raised economic, ethical, and environmental sustainability concerns, reinforcing the need for judicious imaging strategies and dose optimization [6]. Individual cardiac imaging procedures may deliver effective doses ranging from 1 to 60 millisieverts (mSv), with an average reference dose of approximately 15 mSv—equivalent to about 750 chest X-rays—for procedures such as percutaneous coronary intervention, cardiac radiofrequency ablation, multidetector coronary angiography, or myocardial perfusion scintigraphy [3].

Clinical and epidemiological studies in the cardiology population suggest a potential increase in cancer risk associated with cardiac fluoroscopic catheterization [7], particularly in patients exposed to cumulative radiation doses early in life [8], although risk estimates at doses below 100 mSv remain uncertain [9]. These uncertainties are particularly concerning in pediatric populations, where cardiac catheterization has been described as a form of “friendly fire” that may expose children with congenital heart disease to disproportionate long-term radiation risks [10].

Interventional cardiologists and catheterization laboratory staff experience occupational exposure while working in close proximity to radiation sources, with cumulative lifetime doses estimated between 50 and 200 mSv [11,12]. Recent analyses have identified the head of invasive cardiologists as a particularly vulnerable target of professional radiation exposure, underscoring the importance of optimized shielding and procedural awareness [13]. In this context, some observational data suggest a higher incidence of brain cancers in interventional cardiologists, particularly in the left hemisphere, potentially reflecting occupational exposure patterns [11].

There has been growing interest in the potential non-cancer effects of low to moderate radiation doses, particularly concerning the risk of cardiovascular disease and central nervous system disorders, including cerebrovascular disease incidence and mortality, as well as Parkinson’s disease [1,14]. Occupational exposure has also been associated with a higher prevalence of skin lesions, orthopedic disorders, and cataracts among personnel working in interventional cardiology and cardiac electrophysiology, with risks increasing with the duration of radiation exposure [15].

However, epidemiological studies at low doses are constrained by methodological limitations, including heterogeneous endpoints, uncertainties in dose estimation, and limited control of confounding variables, resulting in insufficient evidence to establish causality [16]. Biomarkers, defined as “any measurement reflecting an interaction between a biological system and an environmental agent”, offer the potential to bridge gaps between radiation exposure and health outcomes by reflecting early biological effects and individual susceptibility [17]. A recent systematic review and meta-analysis demonstrated that medical workers exposed to low-dose ionizing radiation exhibit measurable increases in genotoxicity biomarkers, supporting their relevance for occupational risk assessment [18].

This review summarizes current evidence on genetic and epigenetic biomarkers for evaluating radiation exposure from cardiac imaging and interventional cardiology, emphasizing their potential to improve risk assessment and guide personalized protective strategies. By integrating biomarker data with the principles of justification, optimization, and sustainability, such approaches align with contemporary recommendations for safer and more responsible use of medical radiation in cardiovascular practice [6].

## 2. Current State of the Art of Promising Genetic Biomarkers

### 2.1. γ-H2AX to Monitor DNA Damage Induced by Ionizing Radiation

DNA is the primary biological target of IR-induced damage, which occurs either through direct energy deposition or indirectly via reactive oxygen species (ROS) generation [19]. Radiation exposure produces a broad spectrum of DNA lesions, including base damage, apurinic/apyrimidinic sites, single-strand breaks, DNA–protein cross-links, and double-strand breaks [19]. Among these, double-strand breaks represent the most biologically deleterious form of DNA damage, as inaccurate repair may result in chromosomal aberrations, genomic instability, and carcinogenesis [20].

The histone H2A variant H2AX plays a central role in the early cellular response to double-strand breaks, serving as a platform for the recruitment and organization of DNA damage response proteins, thereby contributing to genome stability maintenance [19,20]. Following double-strand break induction, H2AX is rapidly phosphorylated at serine 139 by phosphatidylinositol-3-kinase–related kinases (ATM, ATR, and DNA-PKcs), leading to the formation of γ-H2AX [21]. This phosphorylation extends across megabase regions surrounding the break site and occurs within minutes after exposure, with γ-H2AX levels demonstrating a strong dose-dependent relationship with double-strand break frequency [22].

γ-H2AX can be visualized as discrete nuclear foci using immunofluorescence microscopy, providing a highly sensitive and quantitative method for detecting double-strand breaks within chromatin at doses well below the detection limits of conventional cytogenetic assays [19,23]. It is essential for the retention and stabilization of multiple DNA repair factors, including MDC1, 53BP1, BRCA1, and RAD51, and contributes to regulation of both non-homologous end joining and homologous recombination pathways [19,22].

In vitro studies consistently show that γ-H2AX foci formation peaks approximately 30–60 min after irradiation in a dose-dependent manner, followed by a gradual decline over several hours reflecting double-strand break repair kinetics rather than simple damage disappearance [21,22]. Although initially developed for experimental systems, the γ-H2AX assay has increasingly been applied in vivo to assess DNA damage in individuals exposed to IR during diagnostic imaging and radiotherapy, including cardiac computed tomography and fluoroscopically guided procedures [24].

In pediatric cardiac catheterization, γ-H2AX foci analysis has demonstrated measurable DNA damage even at relatively low X-ray doses, raising concerns that radiation-associated biological risks in young patients may be underestimated [24]. Recent clinical studies have further shown that γ-H2AX foci can be detected in peripheral blood lymphocytes after medical exposures below 10–20 mGy, highlighting its potential utility as a biological dosimeter for low-dose radiation [24,25,26].

Cardiac CT-specific investigations have confirmed significant correlations between γ-H2AX induction and scanner-derived physical dose parameters, supporting its role as a biologically relevant complement to conventional dosimetry [27]. Multi-biomarker approaches combining γ-H2AX with protein and gene expression markers have also demonstrated that cardiac CT angiography induces measurable radiation-related biological responses in circulating lymphocytes [28]. Similarly, nuclear medicine procedures such as cardiac dual-isotope imaging produce quantifiable increases in γ-H2AX foci, confirming DNA damage after moderate diagnostic exposures [29].

However, clinical applicability of γ-H2AX foci in circulating lymphocytes is limited by rapid post-exposure signal decay, restricting the temporal window for sample collection [30]. In contrast, chronic or repeated low-dose exposure, as experienced occupationally by interventional cardiologists, may induce persistent alterations in double-strand break signaling and baseline γ-H2AX levels, potentially reflecting long-term modulation of DNA damage response capacity [24,31]. Studies in interventional cardiology personnel have demonstrated sustained elevations in γ-H2AX and other genotoxicity markers, suggesting cumulative biological effects of occupational low-dose exposure [31].

### 2.2. Radiation-Induced Chromosome Damage: Micronucleus Assay

Micronuclei are small extranuclear bodies formed from acentric chromosome fragments or whole chromosomes that fail to incorporate into daughter nuclei during anaphase, reflecting both clastogenic and aneugenic events [32]. They can be readily identified and quantified using conventional light microscopy or automated image-analysis platforms, enabling both small-scale and high-throughput applications [32,33].

In human biomonitoring studies, micronuclei are typically assessed in peripheral blood lymphocytes during the first post-mitotic interphase, providing a validated and sensitive biomarker of chromosomal damage and genomic instability [32,33].

Peripheral blood lymphocytes, in fact, serve as a valuable in vitro model for studying radiation-induced effects because of their high radiosensitivity, their largely non-proliferative state in vivo, and their widespread use in radiobiological assessments. Their well-characterized response to IR provides critical insights into DNA damage and repair mechanisms, and overall cellular stress responses in normal human cells, making them an essential cell model for both mechanistic studies and translational research. Peripheral blood lymphocytes are particularly suitable for examining radiation-induced chromosomal damage because they are inherently synchronized in the G0 phase of the cell cycle, ensuring consistent radiosensitivity across all cells [34]. Elevated micronucleus frequencies have been observed in cancer, cellular senescence, and following genotoxic exposure, and are considered intermediate endpoints in carcinogenesis [33].

Micronuclei quantified using the cytokinesis-block micronucleus assay show promise as biomarkers of individual radiosensitivity and susceptibility to environmental and occupational carcinogens [32,35]. Consistent with research priorities outlined in the National Academy of Sciences Biologic Effects of Ionizing Radiation (BEIR) VII report, chromosomal biomarkers have increasingly been applied as intermediate carcinogenesis endpoints to assess potential health risks associated with cardiac imaging procedures [36]. Regarding patient exposure to low-dose ionizing radiation, children with congenital heart disease who undergo repeated cardiac catheterizations show sustained elevations in micronucleus frequency on long-term follow-up, indicating persistent chromosomal damage years after radiation exposure [37]. Acute exposure to ionizing radiation during these procedures has also been associated with an immediate increase in chromosomal damage. The median micronucleus frequency increased from 6‰ at baseline to 9‰ at 2 h after the procedure [38]. Elevated micronucleus levels compared with baseline have been reported following both diagnostic and therapeutic cardiac catheterization procedures [39].

Moreover, contemporary interventional cardiologists exhibit significantly higher micronucleus frequencies than non-exposed cardiologists, indicating that occupational exposure to chronic low-dose radiation is associated with increased chromosomal damage [40]. These findings suggest the micronucleus assay may serve as a valuable biological index within occupational surveillance programs. However, longitudinal studies with repeated measurements in larger worker cohorts are required to establish reliability, sensitivity, and predictive value for low-dose exposure assessment.

### 2.3. Changes in Telomere Length and Radiation Exposure

Telomeres are specialized nucleoprotein structures located at the ends of linear chromosomes, composed of tandem TTAGGG repeats bound by the shelterin complex, which protects chromosome termini from recognition as DNA double-strand breaks [41]. With each somatic cell division, telomeres progressively shorten due to the end-replication problem and oxidative stress. Critically shortened telomeres activate DNA damage signaling pathways that induce replicative senescence or apoptosis [41]. Telomere shortening is widely regarded as a marker of biological aging and has been associated with increased risk of cancer, cardiovascular disease, and overall mortality, although causal relationships remain under investigation [42].

Accumulating evidence indicates that telomere integrity plays a significant role in cellular and organismal responses to IR by influencing genome stability and radiosensitivity [43]. Loss of telomere function results in chromosomal end-to-end fusions, breakage–fusion–bridge cycles, and genomic instability, and has been linked to radiation-induced genetic instability and enhanced radiosensitivity [43].

An association between telomere length and individual radiation sensitivity was first described in cells derived from patients with inherited radiation-sensitive syndromes, such as ataxia-telangiectasia and dyskeratosis congenita [44]. Subsequent in vitro studies demonstrated that short telomeres are linked to impaired DNA repair capacity and increased sensitivity to IR [45]. In contrast, human data on telomere dynamics following radiation exposure remain limited. Some studies have reported telomere shortening in peripheral blood leukocytes of cancer patients undergoing radiotherapy, suggesting systemic biological effects even after localized radiation exposure [46,47]. Persistent telomere shortening, together with altered apoptotic responses, has also been observed in blood cells of Chernobyl clean-up workers both shortly after exposure and decades later, even following relatively low-dose irradiation [48].

Concerns about cumulative biological and cardiovascular risks associated with repeated imaging-related radiation exposure have also emerged from prospective coronary heart disease screening using coronary computed tomography angiography in cancer survivors [49]. In patients with congenital heart disease, leukocyte telomere shortening has been documented in adulthood, suggesting that disease-related factors and repeated medical exposures may contribute to accelerated biological aging in this population [50]. More recently, the effects of low-dose radiation exposure in interventional cardiology settings have been investigated. Andreassi et al. demonstrated for the first time that long-term occupational exposure to radiation in catheterization laboratories may be associated with telomere shortening and increased subclinical carotid intima–media thickness, potentially contributing to accelerated vascular aging and the development of atherosclerosis [51].

### 2.4. Mitochondrial Dysfunction After Radiation Exposure

Over the past several decades, remarkable progress has been achieved in the field of human mitochondrial disease, yielding critical insights into the principal pathological pathways that underlie and regulate mitochondrial dysfunction. Recent advances have substantially reshaped our understanding of mitochondrial biology, particularly with regard to the intricate molecular networks that govern mitochondrial function in both physiological and pathological conditions [52].

Owing to its central role in the generation of ROS, the mitochondrial respiratory chain is widely recognized as a major pathogenic contributor to a broad spectrum of disease states. ROS-induced oxidative stress is a well-established driver of cellular senescence and organismal aging, processes that are frequently accompanied by the accumulation of mitochondrial DNA (mtDNA) mutations and progressive energetic decline. This deterioration places mitochondria in a self-perpetuating and deleterious cycle characterized by escalating ROS production and further mitochondrial damage [53].

Although IR has traditionally been understood to injure target cells primarily through the induction of nuclear DNA damage and genomic instability, emerging evidence indicates that many IR-induced biological effects are more closely associated with mitochondrial dysfunction and excessive ROS generation than with other initiating triggers or pathological mechanisms [54]. Multiple investigations have documented elevated endogenous ROS production across a variety of IR-exposed cell line models. Irradiation has been shown to increase mitochondrial superoxide radical generation, a phenomenon strongly linked to mitochondrial dysfunction and to significant alterations in mitochondrial properties, including a reduction in mitochondrial membrane potential and compromised bioenergetic performance [55,56].

Concurrently, additional studies have demonstrated that IR exposure results in extensive DNA damage, including oxidative lesions and double strand breaks (DSBs) within mtDNA, which appears to be more vulnerable to IR-induced oxidative insults than nuclear DNA. Importantly, mtDNA repair systems are comparatively less efficient and less robust than those operating in the nucleus, a limitation that may contribute substantially to the pathogenesis of numerous disorders associated with declining mitochondrial activity and impaired cellular energetics [57]. In this context, evaluation of the mitochondrial-to-nuclear DNA ratio has emerged as an essential parameter for assessing radiation responses and the extent of mitochondrial dysfunction [58]. Furthermore, inherited and acquired genetic variations in mtDNA have been utilized to quantify inter-individual differences in radiosensitivity among exposed populations, underscoring the relevance and translational value of this approach in radiation research [59].

Despite the well-documented evidence that IR-induced damage compromises mitochondrial structural and functional integrity, oxidative stress has also been reported to induce an increase in mtDNA copy number in both in vitro and in vivo experimental systems [58]. This phenomenon, commonly referred to as mitochondrial polyploidization, is thought to represent a compensatory adaptive response to the loss of genetic material and functional capacity caused by oxidative injury. Through this mechanism, mitochondria may attempt to preserve bioenergetic competence and sustain cellular homeostasis following irradiation exposure [56,60].

Increasing attention has been directed toward elucidating the role of mitochondrial dysfunction in radiation-induced heart disease and clarifying its impact on cardiac performance and long-term cardiovascular outcomes [61]. Mitochondria constitute essential organelles within cardiomyocytes, accounting for up to 30% of total cellular volume and reflecting the exceptionally high energetic demands of cardiac tissue. Given these substantial metabolic requirements, efficient mitochondrial respiration is indispensable for maintaining contractile function and overall myocardial viability [62]. In human patients, ultrastructural analyses using electron microscopy have revealed that radiation exposure profoundly disrupts mitochondrial architecture, including disorganization and loss of cristae, a reduction in cristae density, and the induction of mitochondrial swelling. Comparable structural abnormalities have also been documented in rabbit cardiomyocytes following exposure to a single dose of IR, reinforcing the translational relevance of these findings across species [61].

Notably, the frequency of the mtDNA common deletion (4997 bp), widely regarded as a molecular marker of oxidative stress and cumulative mitochondrial damage, is significantly elevated in cardiomyocytes subjected to oxidative stress conditions [63]. In addition, investigations involving occupationally exposed workers have revealed decreased expression levels of key mitochondrial respiratory chain proteins, including complexes I and III, in post-mortem cardiac tissue samples [64]. Moreover, the mtDNA-4977 bp deletion was found to be significantly higher in the peripheral blood of 147 personnel working in high-volume cardiac catheterization laboratories compared to unexposed individuals, highlighting that, beyond the well-recognized nuclear DNA, mtDNA damage may represent an important pathogenetic pathway and a potential therapeutic target for ionizing radiation-induced injury [65].

Experimental animal studies provide further mechanistic support for these observations, demonstrating increased expression of pro-apoptotic signaling molecules following cardiac irradiation. In rat models, irradiation has been shown to induce cardiomyocyte hypertrophy accompanied by a higher proportion of apoptotic nuclei and an increased Bax/Bcl-2 expression ratio, indicative of enhanced apoptotic susceptibility [62,66]. Taken together, these findings underscore the critical importance of achieving a deeper and more comprehensive understanding of mitochondrial involvement in radiation-induced heart disease, which is essential for the identification of reliable biomarkers and the development of targeted therapeutic strategies aimed at mitigating IR-induced cardiac injury.

### 2.5. Circulating Cell-Free DNA, Radiation Exposure, and Disease Susceptibility

Although most DNA in the human body is intracellular, measurable amounts of extracellular nucleic acids circulate in the bloodstream as circulating cell-free DNA [67,68]. Despite more than three decades of research, the precise biological origins of circulating cell-free DNA remain incompletely defined, particularly under non-pathological conditions [67]. Current evidence indicates that apoptosis and necrosis represent primary sources of circulating cell-free DNA, with additional contributions from active secretion and neutrophil extracellular traps [69,70]. Low concentrations are consistently detectable in healthy individuals, whereas markedly elevated levels have been observed in cancer, autoimmune diseases, acute myocardial infarction, and other inflammatory conditions, reflecting increased cellular turnover and tissue injury [69,71].

Data regarding radiation-induced changes in circulating cell-free DNA remain limited, particularly within the low-dose range relevant to diagnostic imaging and occupational exposure [72].

Preclinical studies have demonstrated clear dose-dependent increases in plasma DNA levels following ionizing radiation, supporting its potential role as a biomarker of radiation-induced tissue damage [73]. In murine models, plasma DNA concentrations increased from baseline nanogram-per-milliliter levels to several hundred nanograms per milliliter after irradiation, depending on dose and post-exposure timing [73]. In cancer patients undergoing radiotherapy, serial measurements have revealed dynamic temporal changes in circulating cell-free DNA, often characterized by transient early increases correlating with treatment-induced cell death [73,74]. These early elevations are consistent with acute radiation-induced cellular injury and apoptosis [70,74]. Beyond oncology, circulating cell-free DNA represents, therefore, an attractive biomarker for population-based studies due to its accessibility and sensitivity to tissue damage. Its detection through minimally invasive blood sampling makes it particularly suitable for occupational and epidemiological investigations, including those conducted outside traditional clinical settings [74,75].

Molecular epidemiology seeks to characterize biological processes linking exposure to clinically overt disease, thereby enabling earlier risk stratification. In this framework, circulating cell-free DNA may represent a mechanistic link between radiation-induced cellular damage and individual disease susceptibility, warranting further investigation as a complementary biomarker in radiation research. In this context, and with regard to chronic occupational exposure from interventional cardiology, Borghini and colleagues investigated the impact of chronic low-dose radiation on serum circulating cell-free DNA (ccf-DNA) levels and circulating cell-free mitochondrial DNA (ccf-mtDNA) fragments (mtDNA-79 and mtDNA-230) in personnel working in high-volume cardiac catheterization laboratories, aiming to assess their potential as radiation biomarkers. Interestingly, their results provide evidence that circulating DNA may serve as a relevant biomarker of cellular damage induced by chronic low-dose radiation exposure [76].

## 3. Epigenetic Biomarkers: DNA Methylation and MicroRNAs

Ionizing radiation can induce stable changes in gene expression without altering the underlying DNA sequence, a phenomenon termed epigenetic modification [77]. These alterations are increasingly recognized as contributors to human diseases, including cardiovascular disorders and cancer [77].

Epigenetic changes may impair DNA repair capacity by silencing repair genes through promoter hypermethylation or altering chromatin accessibility, thereby increasing radiosensitivity and genomic instability [78]. Major epigenetic mechanisms include DNA methylation, histone modifications, chromatin remodeling, and regulation by non-coding RNAs such as microRNAs (miRNAs). DNA methylation, the most extensively studied epigenetic modification, involves covalent addition of a methyl group to the fifth carbon of cytosine residues, predominantly within CpG dinucleotides located in gene promoters or regulatory regions CpG islands, frequently present in promoters of housekeeping and many tissue-specific genes, strongly influence transcriptional activity according to their methylation status. Promoter hypermethylation generally represses transcription, whereas hypomethylation may promote aberrant activation of oncogenes or pro-inflammatory pathways [78].

Ionizing radiation has been shown to induce global hypomethylation, particularly of repetitive elements, together with promoter hypermethylation of tumor-suppressor genes, thereby contributing to radiation-induced genomic instability [79]. Low-dose exposure from computed tomography has been associated with detectable alterations in DNA methylation patterns in peripheral blood cells concomitant with double-strand break formation [80].

MiRNAs are short (19–25 nucleotides), highly conserved non-coding RNAs that regulate gene expression post-transcriptionally and represent an expanding class of epigenetic regulators in radiation biology. Their expression profiles change dynamically in response to ionizing radiation, and many radiation-responsive miRNAs regulate DNA repair, apoptosis, and cell-cycle control pathways, thereby modulating radiosensitivity [81].

DNA repair processes can also influence miRNA expression, creating feedback loops in which the nature and extent of DNA damage determine specific miRNA activation or suppression, thereby fine-tuning repair efficiency [82]. MiRNAs regulate double-strand break repair by targeting key proteins, including H2AX [82].

miR-138 and miR-24 have been functionally implicated in H2AX regulation [83,84] miR-138 was identified through high-throughput screening using γ-H2AX foci formation as a readout and acts as a negative regulator of DNA damage signaling [83]. miR-24 overexpression suppresses H2AX, increasing hematopoietic cell sensitivity to gamma irradiation and potentially explaining reduced repair capacity in terminally differentiated cells [84]. Additionally, miR-27a modulates ATM kinase expression, influencing early double-strand break repair kinetics, whereas miR-185 downregulation after irradiation and its overexpression enhance apoptosis by targeting ATR kinase [81].

In patients exposed to low-dose radiation from cardiac scans, altered expression of miRNAs such as miR-21 and miR-625 has been documented, supporting their in vivo relevance [85]. A significant association between pediatric radiation exposure from cardiac procedures and alterations in early markers of genetic instability and carcinogenesis, including dysregulation of miR-155, a well-known oncogenic miRNA, was also observed [86]. miR-155 promotes tumor growth, angiogenesis, and inhibition of apoptosis. Notably, it is overexpressed in cells from patients with acute myeloid leukemia and in several solid tumors arising in highly radiosensitive organs, such as the lung, breast, and colon [87].

Regarding the occupational exposure, interventional cardiologists chronically exposed to low-dose radiation have been found to exhibit deregulation of miRNA-134 and miRNA-2392 in peripheral blood [88]. This study revealed that circulating brain miR-134 and miR-2392 expression profiles were significantly downregulated in interventional cardiologists compared with controls [88].

Although the exact function of miR-2392 remains unclear, recent evidence indicates that it is downregulated in gastric cancer cell lines and tissues, and its overexpression inhibits tumor cell invasion and metastasis both in vitro and in vivo [89]. miR-134 was first identified as a brain-specific miRNA involved in synapse development and directly implicated in learning and memory. It has been previously reported as being dysregulated in mesial temporal lobe epilepsy, Alzheimer’s disease, bipolar disorder, oligodendrogliomas, and glioblastomas [90]. The pronounced dysregulation of the brain-specific miR-134 strongly suggests that brain damage may represent a major long-term risk of unprotected head irradiation in interventional cardiologists, with potential long-lasting consequences for cognitive function.

**Figure 1:**
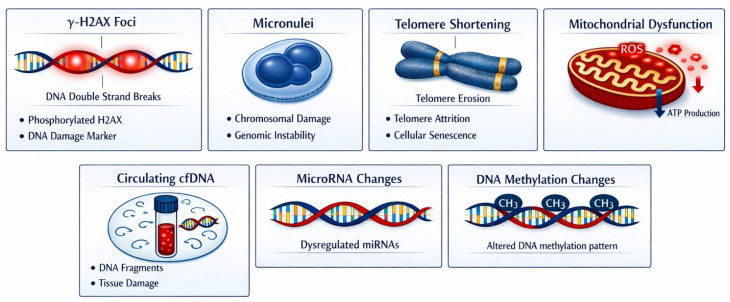
Genetic and epigenetic biomarkers of low-dose ionizing radiation exposure in cardiac imaging. Image generated with AI Microsoft Copilot 365 (GPT-5) Image generator.

Longitudinal studies evaluating persistent epigenetic alterations remain absent, highlighting a significant research gap, particularly in occupational and medical low-dose exposure settings. Figure 1 summarizes current evidence on genetic and epigenetic biomarkers for evaluating the biological effects of low-dose IR exposure in cardiac imaging.

## 4. Conclusions and Future Directions

Identifying reliable biomarkers of radiation response remains a major scientific priority. The BEIR VII report emphasized the need for research on molecular and functional biomarkers in occupational and medical radiation exposure contexts [32]. The United Nations Scientific Committee on the Effects of Atomic Radiation (UNSCEAR) recommended integrated studies combining clinical, subclinical, and molecular endpoints to clarify health effects in exposed populations [91].

Genetic and epigenetic biomarkers provide promising tools to detect and quantify biological damage after low-dose radiation exposure from cardiac imaging and may enable early identification of high-risk individuals. However, the clinical and translational use of these responsive biomarkers is limited by their heterogeneity in temporal dynamics, persistence, and susceptibility to confounding factors. Early markers such as γ-H2AX are highly sensitive but only suitable for acute exposure due to their rapid decline, whereas cytogenetic markers like the micronucleus assay provide longer-term information but lack specificity. Similarly, markers of chronic exposure, such as mitochondrial DNA damage and telomere length, reflect long-term effects but show high inter-individual variability and are influenced by non-radiation factors. Epigenetic biomarkers can detect stable changes but are complex to interpret and also affected by environmental and endogenous influences (Table 1).

Overall, no single biomarker fully meets all the criteria of an ideal radiation biomarker. Current evidence supports a multi-biomarker approach that integrates early and late indicators to better capture both acute and long-term effects. A key challenge, especially at low doses, is distinguishing radiation-induced signals from other biological or environmental influences, highlighting the need for longitudinal studies, individual dosimetry, and combined biomarker panels to improve specificity.

**Figure 2:**
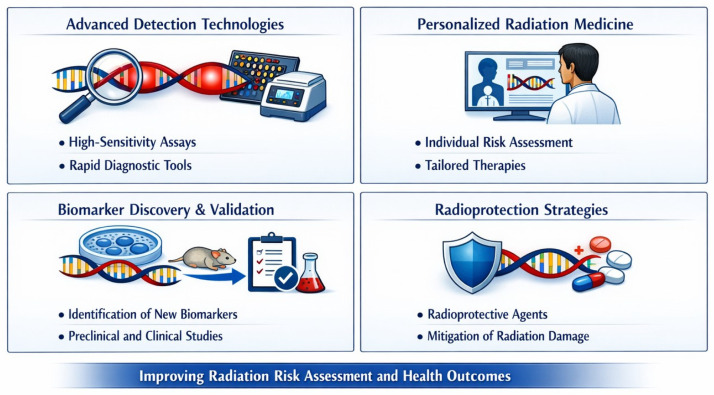
Schematic overview of emerging priorities in radiation-biomarker research, spanning next-generation detection technologies, integration of biomarker signatures into precision radiation medicine, structured pipelines for discovery and validation across model systems and clinical cohorts, and the development of radioprotective strategies to enhance risk assessment and mitigate long-term health effects. Image generated with AI Microsoft Copilot 365 (GPT-5) Image generator.

Future large-scale prospective studies should rigorously evaluate diagnostic, prognostic, and predictive value. Integration into clinical workflows could support personalized surveillance and optimized radiation protection strategies. Longitudinal monitoring using γ-H2AX, micronuclei, telomere length, circulating cell-free DNA, mitochondrial dysfunction and epigenetic markers may capture cumulative exposure and inter-individual variability. Combining biomarker assessment with advanced dosimetry would enhance mechanistic understanding and strengthen the link between exposure and clinically relevant outcomes. These strategies also have implications for occupational safety, supporting evidence-based exposure limits and preventive measures for interventional cardiologists and radiology personnel (Figure 2).

## Figures and Tables

**Table 1:** Biomarkers of Ionizing Radiation Exposure: Characteristics, Applications, and Limitations. This table summarizes key biomarkers used to assess exposure to IR, highlighting their biological targets, temporal dynamics, and practical applications.

Biomarker	Biological Target	Persistence	Best Use	Key Limitations
γ-H2AX	DNA double-strand breaks	Hours (rapid decay)	Acute exposure, biological dosimetry	Narrow time window; influenced by other DNA damage sources
Micronucleus assay	Chromosomal damage	Days–years	Retrospective exposure, cumulative damage	Affected by age, smoking, inflammation
Telomere length	Chromosome instability, aging	Days–years	Chronic exposure, aging effects	Strong confounding (age, disease, lifestyle)
Mitochondrial DNA damage	Oxidative stress, mtDNA integrity	Days–years	Chronic exposure	Influenced by metabolic and oxidative conditions
Circulating cell-free DNA	Cell death (apoptosis/necrosis)	Hours–days	Acute tissue damage	Elevated in many diseases
DNA methylation	Gene regulation	Potentially long-term	Long-term exposure effects	Complex interpretation, environmental sensitivity
microRNAs	Post-transcriptional regulation	Days–weeks	Dynamic response profiling	Not radiation-specific, context-dependent

## Data Availability

No new data were created or analyzed in this study. Data sharing is not applicable to this article.

## Abbreviations

The following abbreviations are used in this manuscript:

ccfDNA	Circulating cell-free DNA
DSB	Double-strand break
γ-H2AX	Phosphorylated histone H2AX
IR	Ionizing radiation
mSv	milliSievert
miRNA	MicroRNA
mtDNA	Mitochondrial DNA
MN	Micronucleus
ROS	Reactive oxygen species
